# “Think. Check. Submit.” to avoid predatory publishing

**DOI:** 10.1186/s13054-018-2244-1

**Published:** 2018-11-14

**Authors:** Andrea Cortegiani, Steven L. Shafer

**Affiliations:** 1 Department of Surgical, Oncological and Oral Science (Di.Chir.On.S.). Section of Anesthesia, Analgesia, Intensive Care and Emergency. Policlinico Paolo Giaccone. University of Palermo, Italy, University of Palermo, Via del vespro 129, 90127 Palermo, Italy; 2 Department of Anesthesiology, Perioperative and Pain Medicine, Stanford University, Stanford, CA USA

Fraudulent open access publishing is one of the most serious threats to the scientific community [1]. More than half a million articles have been published in so-called “predatory journals”, which apply charges to the authors without providing (sometimes any!) rigorous editorial services [1]. Email spamming, false metrics, exploiting academic identities for fake editorial board positions, and false peer review processes are frequent practices among these journals (Table 1). Predatory publishers and journals attempt to deceive potential authors by offering websites that appear legitimate, editorial boards populated by prestigious investigators, and evidence of indexing in major databases. Young investigators, eager to “publish or perish”, may fail to recognize that such journals are fundamentally fraudulent. Others may turn to such journals if they become frustrated by the tough, long, and sometimes painful slog of peer review required by legitimate journals [2].

**Table 1:** Common sentences in spam emails from predatory journals

Dear Prof. *Greetings from Journal of…..!!*	
*Based on your research area and previous publications in the relative field, we cordially welcomes you for the Upcoming Issue of...*	
*We understand your busy schedule and request you to submit a case report, a short communication or a mini-review with 500 to 900 words…*	
*We would like to appreciate your contribution towards the scientific community by publishing your precious work. We have gone through your article and found it very knowledgeable.*	
*We’d be truly gratified if you could share your exploration as a Research article, Review article, Case report, Short communication, Conference proceeding or a Thesis with the Journal.*	
*Taking your academic background and rich experience in this field into consideration, the Editorial Board believe that you may be the most suitable candidate for this position.*	
*As we feel that the scope of your research falls under our Journal. With good minds, I am cordially inviting eminent authors like you for article submission.*	

Predatory publishing has been recently surveyed in the fields of anesthesiology, critical care, and emergency medicine [2]. More than 200 potential or probable predatory journals and 80 publishers were found, comprising 12,871 published articles. The mean author charge per article was US$634.50. Almost half of the reported journals’ office locations were unreliable (e.g., supermarkets, highways, football fields, postal boxes) and many journals reported false listings with the Committee of Publication Ethics (COPE) [3], International Committee of Medical Journals Editors (ICMJE) databases, the Directory of Open Access Journals (DOAJ) [4], or Google Scholar. Only roughly 30% of the journals reported the name of the Editor-in-Chief. Rules for ethics, retraction, and editorial flow are rarely reported. The results were similar to other biomedical specialties, such as neurology, nursing, and dermatology. Of note, six journals were indexed in PubMed.

Recently, the “Think. Check. Submit.” campaign has been launched to “help researchers identify trusted journals for their research” [5]. The campaign consists of a checklist that guides researchers through a simple process for assessing the credentials of journals and publishers. Think. Check. Submit. has been produced with the support of several scientific organizations (i.e., COPE, DOAJ, Association of European Research Libraries) and legitimate publishers (i.e., BioMed Central, Springer, Nature). It is available in several languages.

Although resources are now available to avoid predatory journals, many aspects of predatory publishing should be further investigated. For instance, feedback from the authors and editorial board members are not available so we can only speculate on the reasons why researchers decided to submit manuscripts to these journals. Meanwhile, education on the risks to authors, investigators, and patients from supporting predatory publication seems the most effective cure.
